# Introducing an expanded CAG tract into the huntingtin gene causes a wide spectrum of ultrastructural defects in cultured human cells

**DOI:** 10.1371/journal.pone.0204735

**Published:** 2018-10-17

**Authors:** Ksenia N. Morozova, Lyubov A. Suldina, Tuyana B. Malankhanova, Elena V. Grigor’eva, Suren M. Zakian, Elena Kiseleva, Anastasia A. Malakhova

**Affiliations:** 1 Federal Research Center Institute of Cytology and Genetics, Siberian Branch of Russian Academy of Sciences, Novosibirsk, Russia; 2 Department of Natural Sciences, Novosibirsk State University, Novosibirsk, Russia; 3 E.Meshalkin National Medical Research Center of the Ministry of Health of the Russian Federation, Novosibirsk, Russia; 4 Institute of Chemical Biology and Fundamental Medicine, Siberian Branch of Russian Academy of Sciences, Novosibirsk, Russia; Hokkaido Daigaku, JAPAN

## Abstract

Modeling of neurodegenerative diseases *in vitro* holds great promise for biomedical research. Human cell lines harboring a mutations in disease-causing genes are thought to recapitulate early stages of the development an inherited disease. Modern genome-editing tools allow researchers to create isogenic cell clones with an identical genetic background providing an adequate “healthy” control for biomedical and pharmacological experiments. Here, we generated isogenic mutant cell clones with 150 CAG repeats in the first exon of the huntingtin (*HTT*) gene using the CRISPR/Cas9 system and performed ultrastructural and morphometric analyses of the internal organization of the mutant cells. Electron microscopy showed that deletion of three CAG triplets or an *HTT* gene knockout had no significant influence on the cell structure. The insertion of 150 CAG repeats led to substantial changes in quantitative and morphological parameters of mitochondria and increased the association of mitochondria with the smooth and rough endoplasmic reticulum while causing accumulation of small autolysosomes in the cytoplasm. Our data indicate for the first time that expansion of the CAG repeat tract in *HTT* introduced via the CRISPR/Cas9 technology into a human cell line initiates numerous ultrastructural defects that are typical for Huntington’s disease.

## Introduction

Huntington’s disease (Huntington’s chorea, HD) is a severe autosomal dominant disease caused by an increase in the number of CAG (cytosine-adenine-guanine) trinucleotide repeats in the first exon of the huntingtin (*HTT)* gene. The mutant HTT protein that is expressed from the gene with more than 35 repeats leads to death of brain cells, which causes impairment of motor and cognitive functions. Even though a mutation in the *HTT* gene was described more than 20 years ago [1], the molecular and cellular mechanisms of HD are still largely unclear. The pathogenesis of HD has been shown to involve impairment of mitochondrial function [2–4], Ca^2+^ homeostasis [5], and autophagy [6]. Many factors contributing to HD have not yet been determined. Adverse changes in the functions and in interactions of neuronal organelles in HD have also been observed [7, 8]. Medium spiny neurons of the striatum undergo pathological processes at the first stage of disease development, and these processes then spread to other parts of the brain [9]. Studies on mutant neurons have revealed significant disturbances in the structure and dynamics of mitochondria and in their contacts with endoplasmic reticulum (ER) membranes; these problems lead to impairment in calcium ion homeostasis as well as in autophagy and particularly mitophagy [10–12]. Elucidation of the influence of *HTT* mutation on the fine organization of cells and intracellular organelles, such as mitochondria, ER cisternae, and components of the autophagic system, remains one of the essential issues in the HD pathology research.

To understand the successive stages of development of neurodegenerative diseases under the influence of mutant proteins and to search for possible drug targets, both model animals reproducing the pathological phenotype of the disease and neuronal cell models based on patient-specific induced pluripotent stem cells (iPSCs) are currently used [13]. Nonetheless, the results obtained via the patient-specific cell-based approach are significantly influenced by the genetic background of a cell line under study [14, 15]. More promising is the creation of cellular models based on isogenic lines of human cells carrying relevant mutant alleles of the *HTT* gene. Advances in genome-editing technologies based on the CRISPR/Cas9 system give investigators an opportunity to create isogenic cell clones differing only in allelic variants of a target gene [16, 17].

In the present study, we investigated the ultrastructure of human cells of three isogenic mutant clones with deletions or insertions in the *HTT* gene. The mutant cell clones were obtained for the first time via introduction of an HD-causing mutation by the CRISPR/Cas9 technology. A comprehensive analysis by electron microscopy showed that deletion of three CAG repeats or a functional *HTT* knockout by means of a reading frame shift had practically no effect on morphology of the cells, whereas an increased number of CAG repeats caused significant disturbances in the organization of the envelope, cristae, and matrix of mitochondria; stimulated their contacts with ER membranes; and increased the number of autolysosomes and their anomalous variants in the cytoplasm. Morphometric analysis confirmed the structural alterations observed in the mutant cells.

## Materials and methods

### Generation of the HTT-mutant isogenic cell clones

**Fig 1:**
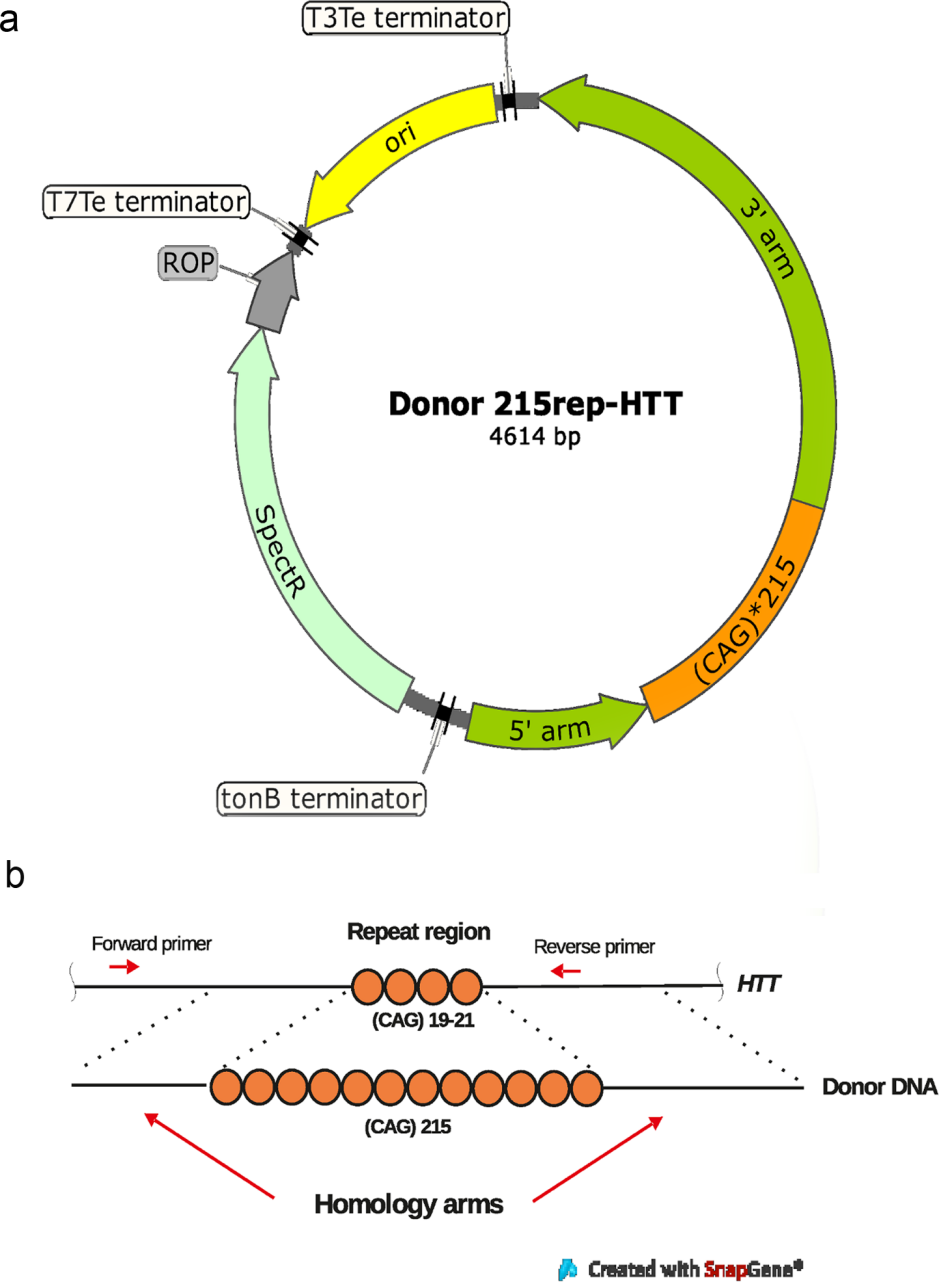
Introducing an expanded CAG repeat tract into the first exon of *HTT* by homology-directed repair. (a) A map of the donor 215rep-HTT plasmid. (b) The scheme of insertion of the expanded trinucleotide tract.

The isogenic cell clones were created from the HEK293 Phoenix cell line by editing the genome with the CRISPR/Cas9 system [18]. The HEK293 Phoenix cell line was kindly provided by Dr. Maria Lagarkova. HEK293 Phoenix cells were cultivated in a medium consisting of Dulbecco’s modified Eagle’s medium (DMEM)/F12 (1:1), 10% of fetal bovine serum, 200 mM GlutaMAX (Life Technologies, USA), and 1% of a penicillin/streptomycin solution (Life Technologies, USA). We designed a 20 nt single guide RNA (sgRNA) sequence (5′-CGTGAGGGAGCGGGGCTGAA-3′) to cut the genomic DNA 19 bases upstream of the CAG repeat tract in *HTT* exon 1 and inserted it into the pSpCas9(BB)-2A-GFP (pX458) plasmid (Addgene, USA) [19]. HEK293 Phoenix cells were cotransfected with both the CRISPR/Cas9-encoding plasmid and donor construct (Fig 1A). The presence of the *EGFP* marker gene in the pX458 plasmid allowed for sorting of transfected cells by green fluorescence on Cell Sorter S3e (Bio-Rad, USA). Thus, single-cell clones of EGFP-positive cells were obtained. Genomic DNA was isolated from cell clones growing in 96-well plates using the Quick-DNA 96 Kit (ZymoResearch, USA). Length of the mutant *HTT* allele was analyzed by PCR (65 mM Tris-HCl pH 8.9, 16 mM (NH_4_)_2_SO_4_, 1.5 mM MgCl_2_, 0.05% Tween 20, 3% glycerol, 6% DMSO, 0.5 U Taq polymerase, primers 0.2 μM each, 50–200 ng genomic DNA)on a C1000 Touch Thermal Cycler (Bio-Rad, USA) (96°C 5 min; 40 cycles: 96°C 20 s, 72°C 3 s; additional synthesis 72°C 15 min) using primers HTT-F 5′-CCCAAGGCCACCTCGGCTCAGAGTC-3′ and HTT-R 5′-CGCAGGCTGCAGGGTTACCGCCATC-3′.

PCR products synthesized from total genomic DNA were sequenced (BigDye Terminator v3.1 Cycle Sequencing Kit, Thermo Fisher Scientific, USA) and analyzed in sequence trace decomposition software TIDE (https://tide-calculator.nki.nl/, [9]) to reveal open reading frame (ORF)-shifting mutations.

Immunofluorescent staining with an anti-huntingtin (N-terminal) antibody (Sigma, H7540, Germany) was performed as described earlier [20].

HTT expression was confirmed by RT-PCR using primers 5′-GGATGGCGGTAACCCTGCAG-3′ and 5′-GATCAGACTCTCCCTTCTCCCGAC-3′ [18]. Cell lysates for western blotting were prepared in RIPA buffer (Thermo Fisher Scientific, USA). Cell lysate protein concentrations were determined by the BCA protein assay (Thermo Fisher Scientific, USA). Lysates (40 μg) were prepared in 4×SDS-PAGE loading buffer (200 mM Tris-HCl, pH 6.8, 400 mM DTT, 8% SDS, 0.4% bromophenol blue, 40% glycerol). The samples were boiled for 5 min at 98°C and separated by one-dimensional SDS-PAGE on a 7.5% acrylamide gel at 100 V for 4 h in running buffer (25 mM Trisbase, 3.5 mM SDS, 200 mM glycine) on ice. Overnight transfer was performed at constant voltage 30 V for 16 h onto an Immun-Blot PVDF membrane (Bio-Rad Laboratories, USA) in transfer buffer (25 mM Trisbase, 192 mM glycine, 10% ethanol) at 4°C. After blockage in 5% milk in TBST, a primary antibody (Sigma Anti-Huntingtin N terminus (3–16), H7540, 1:4000; Bethyl, anti-SMC1 antibody, A300-055A, 1:4000) was incubated with the membranes overnight at 4°C. The membranes were incubated with a secondary antibody (HRP-conjugated anti-rabbit IgG antibody; 1:10000, Jackson ImmunoResearch) at room temperature for 2 h in the blocking solution. Protein bands were detected by chemiluminescence with the Pierce ECL Western Blotting Substrate (Thermo Fisher Scientific, USA).

### Proliferation and viability assay

The proliferation rates of the mutant clones were estimated using real-time cell analyzer iCELLigence (ACEA Biosciences, USA). The cells were seeded in electronic microtiter plates (E-Plates, ACEA Biosciences, USA) at 5 × 10^4^cells per well and analyzed on the RTCA iCELLigence (ACEA Biosciences, USA).

The viability of mutant cell clones cultured under standard conditions was evaluated using the ApoDETECT Annexin V-FITC Kit (ThermoFisher Scientific, USA). The assay was performed in triplicate and analyzed by Wilcoxon’s test.

### Electron microscopy

For electron microscopy, the cells were seeded at 5×10^5^/well in a 12-well plate. Cells growing in culture on the Melinex polyester film (175 μm thickness, Agar Scientific, UK) were fixed in 2.5% glutaraldehyde in the culture medium for 15 min, followed by fixing with 2.5% glutaraldehyde in 0.1 M sodium cacodylate buffer (pH 7.3) for 1 h at room temperature. The samples were washed three times in the same buffer and additionally fixed in 1%osmium tetroxide for 1 h, washed twice in double-distilled (dd) H_2_O, and incubated in 1% uranyl acetate for 12 h at 4°C. Next, the samples were dehydrated in a graded series of ethanol solutions (from 30% to 100%, 10 min in each) and then acetone (twice, 10 min), were embedded in epoxy resin Epon 812 (Sigma, USA), and polymerized for 2 days at 60°C [21, 22]. The cells remained attached to the Melinex film for all treatments and embedding. The most cell-enriched areas were marked on the polymerized plates; then, a block with a diameter of approximately 2 mm was cut out. Ultrathin sections for transmission electron microscopy analysis (50 nm thickness) were cut off in parallel to the plane of the substrate by means of a diamond knife on a Leica EM UC7 ultramicrotome (Leica, Austria).The sections were examined and photographed under a transmission electron microscope, JEOL-1400 (JEOL, Japan), with a Veleta camera (Olympus, USA) and iTEM 5.1 software (Olympus, USA).

### Morphometric analysis

Quantitative parameters of the organelles (autolysosomes and mitochondria) of the cells were evaluated on randomly selected sections obtained from three randomly selected areas of embedded samples. The operator was blinded to the assignment of sections to experimental groups. The proportions of mitochondria with various impairments as well as those in tight contact with ER membranes and other mitochondria were calculated as the percentage of the corresponding types of organelles in cell cytoplasm on the sections. In total, 60 randomly selected cells and approximately 600 mitochondria were examined for each cell clone. The relative number and volume density of autolysosomes were determined as the number of organelles or their area per 1 μm^2^ area of the cytoplasm in a cell section [22]. Autolysosomes were conventionally subdivided into two groups: small organelles having a maximum diameter of 0.1 to 0.6 μm and large ones 0.7 to 2 μm or larger. The measurements were carried out in the ImageJ freeware (ImageJ, USA, https://imagej.nih.gov/ij/). Significance of the differences in the compared mean values was verified by one-way ANOVA followed by Fisher’s multiple-comparison test in the SPSS Version 11.0 statistical software package (IBM Corp., Armonk, NY, USA).

## Results

### Characterization of HEK293 cell lines harboring mutations in the *HTT* gene

#### Creation of mutant HEK293 cell clones using the CRISPR/Cas9 genome-editing tool

**Fig 2:**
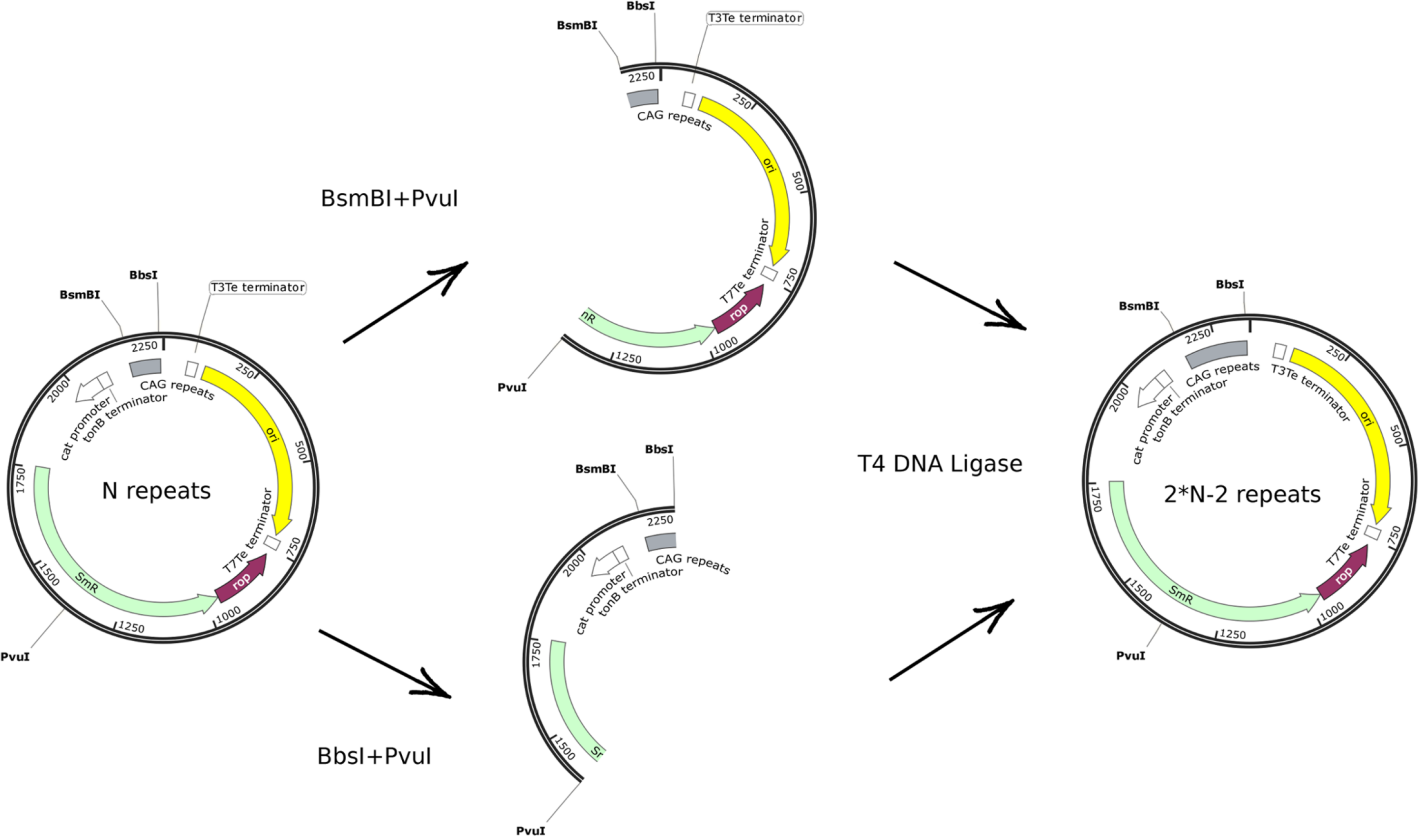
Generation of the expanded CAG repeat tract by the golden gate cloning method. The design of the experiment.

Using the genome-editing method based on the CRISPR/Cas9 system, we obtained a panel of isogenic cell clones with an identical genetic background differing in the number of CAG repeats in the *HTT* gene. An expanded CAG repeat tract was introduced into the first exon of *HTT* by homology-directed repair (Fig 1B) [19]. First, we generated a donor construct bearing 215 CAG trinucleotide repeats flanked with long homology arms (Fig 1A). Oligonucleotides containing three CAG repeats and recognition sites for type SII restriction enzymes, BsmBI and BbsI, were synthesized. The long trinucleotide repeat tract was obtained by repetitious rounds of digestion/ligation by the golden gate cloning method (Fig 2) as described elsewhere [18]. We used the pSMART cloning vector (Lucigen, USA) and the recA13Stbl3 *E*. *coli* strain, which are designed specifically for cloning direct repeats to reduce the frequency of recombination of long repetitive DNA tracts.

Oligonucleotides containing three CAG repeats and recognition sites for type SII restriction enzymes, BsmBI and BbsI, were synthesized and introduced into the pSMART cloning vector. The plasmid was digested with restriction enzyme pairs PvuI + BbsI and PvuI + BsmBI. The plasmid fragments containing CAG repeats were then isolated and ligated to each other. The trinucleotide repeat sequence was elongated by means of repeated rounds of digestion–ligation according to the formula X = 2N–2, where N is the number of the digestion–ligation rounds. X is the resulting number of the CAG repeats.

The 5′ and 3′homology arms were amplified from genomic DNA using the Phusion High-Fidelity PCR Master Mix with GC Buffer and introduced along with the expanded CAG tract into a donor plasmid (donor 215rep-HTT; Fig 1B). The donor construct served as a template for homologous recombination.

HEK293 Phoenix cells were cotransfected with both the CRISPR/Cas9 encoding plasmid (pX458-HTT) and the donor construct. The presence of the *EGFP* marker gene in the pX458 plasmid allowed for sorting of transfected cells. Over 100 single-cell clones of EGFP-positive cells harboring both the expanded trinucleotide tract and deletions in *HTT* exon 1 were obtained. The length of the mutant *HTT* allele was analyzed by PCR (S1 Fig) [4]. It was shown that only a truncated tract of 215 CAG repeats was introduced into the genome of cells during the homology-directed repair. It should be noted that some of the clones had more than two *HTT* alleles of different length because the HEK293 cells have a hypotriploid karyotype as confirmed by fluorescence *in situ* hybridization (FISH) analysis with a human chromosome 4 painting probe (S2 Fig). It was revealed that the HEK293 Phoenix cells have two full-length copies of chromosome 4 and show a translocation of an additional small fragment of the chromosome 4 short arm.

Cell clones with insertion into the *HTT* gene (6H cells) and two clones with deletions (8D and 8H cells) were subjected to sequencing, and the results were analyzed in sequence trace decomposition software TIDE (https://tide-calculator.nki.nl/, [9]) to confirm the ORF shifts (S3 Fig) [4, 5].

It was found that the 8D cell clone has a deletion of three CAG triplets in the first exon of the *HTT* gene, the 8H clone bears a deletion causing an ORF shift, clone 6H has a normal allele and insertions of 100 and 150 CAG repeats into the gene (Table 1). The ORF-shifting mutations are caused by the CRISPR/Cas9 retrial digest during the genome-editing procedure [6].

**Table 1:** Genotypes of the mutant clones of HEK293 cells harboring the *HTT* gene with introduced mutations.

Cell clone	*HTT* mutation	*HTT* ORF
**HEK293 Phoenix (original cell line)**	Normal alleles	Maintained
**8D**	9 bp [(CAG)_3_] deletion in exon 1	Maintained
**8H**	98 bp deletion	Shifted
**6H**	300 and 450 bp insertion	Maintained

#### The HTT expression assay

The *HTT* gene is known to be widely expressed in different types of human tissues; therefore, we examined the mutant-HTT expression in the mutant clones (S1 Fig). The immunofluorescent assay revealed cytoplasmic localization of the HTT protein, with a slightly increased HTT concentration in the vicinity of the nuclear envelope. No aggregates of mutant HTT were detected (S1 Fig).

#### The viability and proliferation assay

**Fig 3:**
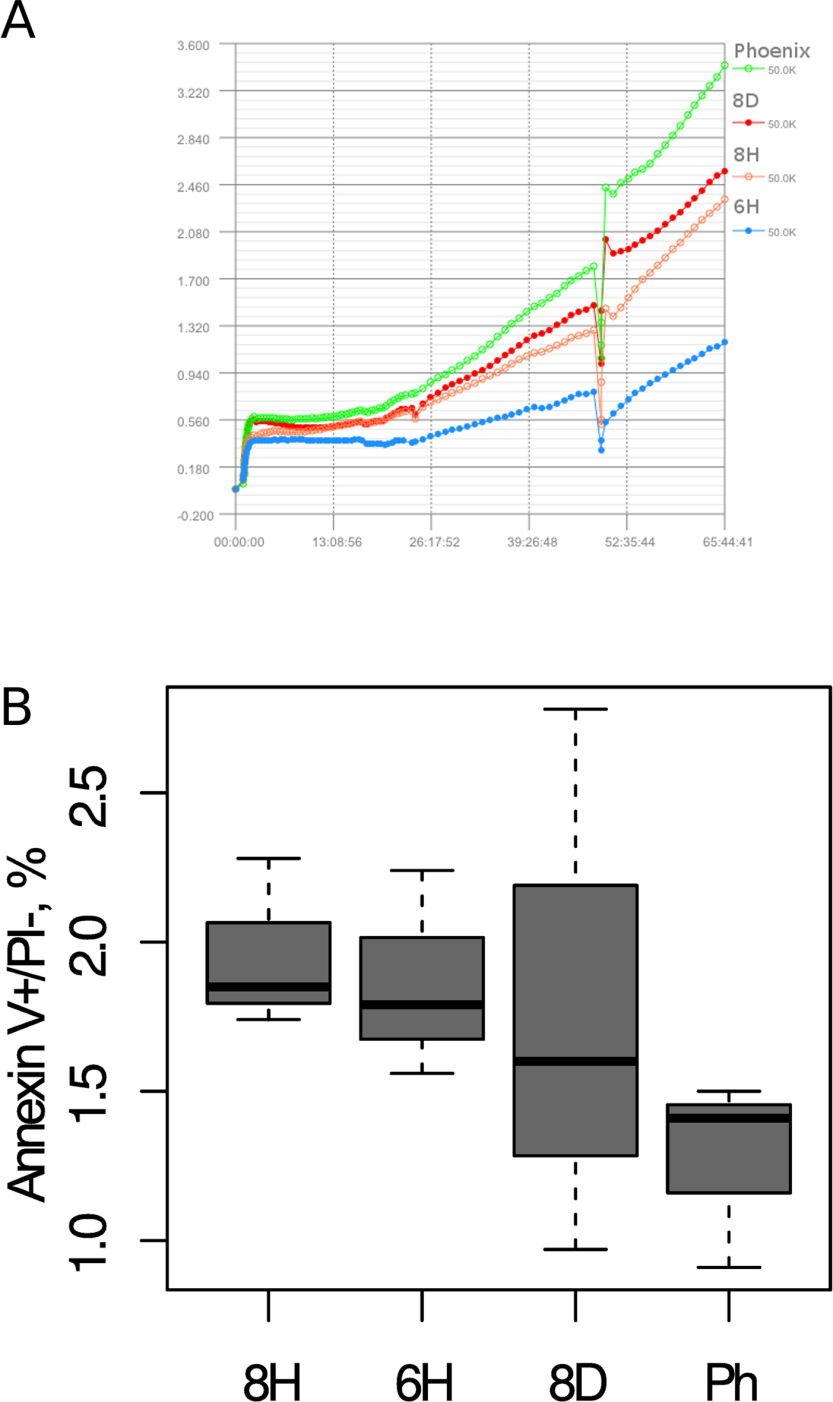
**Assessment of the proliferation rate (a) and viability (b) of three mutant clones (8D, 8H, and 6H) compared to the original HEK293 cell line.** (a) The proliferation assay of mutant clones was conducted by real-time cell analysis. The negative peak in the plot (a) is the consequence of medium replacement (approximately 48 h after the start of the experiment). (b) Apoptosis intensity in mutant cell clones was estimated by an annexin V/propidium iodide cell viability assay. Data scatter of early apoptotic cells (annexin V positive / PI negative) is presented. The bars show standard deviation, n = 3.

Given that HEK293 cells express some neural markers that correlate with certain processes occurring in neural tissue [23, 24], we decided to estimate viability and proliferation rates of the three mutant clones (8D, 8H, and 6H) compared with an isogenic control cell line. We measured proliferation rates of the three mutant clones compared to the original HEK293 Phoenix cell line by real-time cell analysis. The 6H clone has the slowest growth rate as compared to the other two clones and the original HEK293 Phoenix cell line (Fig 3A). Increased apoptosis in mutant clones 6H and 8H compared with HEK293 Phoenix cells was revealed by an annexin V/propidium iodide cell viability assay (Fig 3A).

### Ultrastructure of cultured HEK293 cells (control)

**Fig 4:**
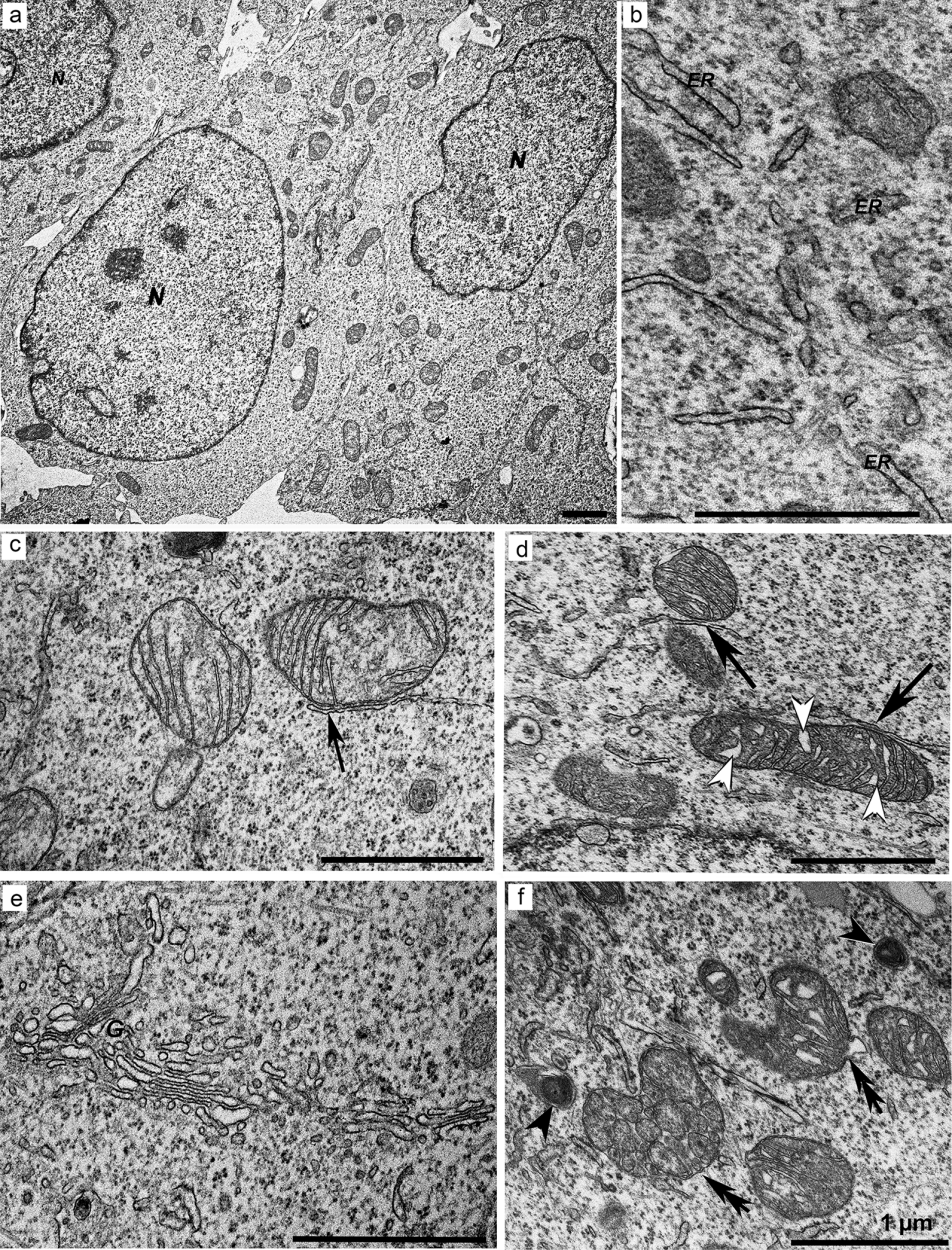
Ultrastructure of the control HEK293 Phoenix cell line. (a) HEK293 Phoenix cells (an overview). (b) Short fragments of the cisternae of the rough ER (*ER*). (c) Mitochondria with a light matrix and narrow parallel cristae. (d) Mitochondria with a dense matrix and narrow dilated cristae (*white arrowheads*). *Black arrows* in (c) and (d) indicate ER membranes in close proximity to mitochondria. (e) An actively functioning Golgi complex forming a large number of vesicles. (f) Defective mitochondria with abnormal structure (indicated with *double arrows*) and autolysosomes (*arrowheads*). *N*: nucleus, *G*: Golgi complex. *Scale bars*: 1 μm.

The cells had an oval shape, large nuclei with electron-dense nucleoli, and some invaginations of the nuclear envelope (Fig 4A). The cytoplasm contained a large number of polysomes, mitochondria, and short rough and smooth ER cisternae (Fig 4B). Mitochondria in cells were either dispersed or grouped and could be classified into two types by morphology. The first type contained a light matrix and narrow parallelly oriented cristae (Fig 4C), whereas the second type had a denser matrix and swollen cristae (Fig 4D). It was observed that certain mitochondria came into contact with ER cisternae (Fig 4C and 4D). Cells in the population could contain either both types of mitochondria or one of them predominantly. Some mitochondria had irregular arrangement of cristae together with low density of the matrix. The cytoplasm contained a well-developed Golgi complex consisting of cisternae and vesicles of various sizes (Fig 4E). Autolysosomes of various diameters surrounded by one limiting membrane and some disintegrating mitochondria with destroyed cristae were also identified (Fig 4F).

### Ultrastructure of mutant cell clones harboring a deletion in the *HTT* gene (clone 8D) or an *HTT* gene with a shift of the reading frame (clone 8H)

**Fig 5:**
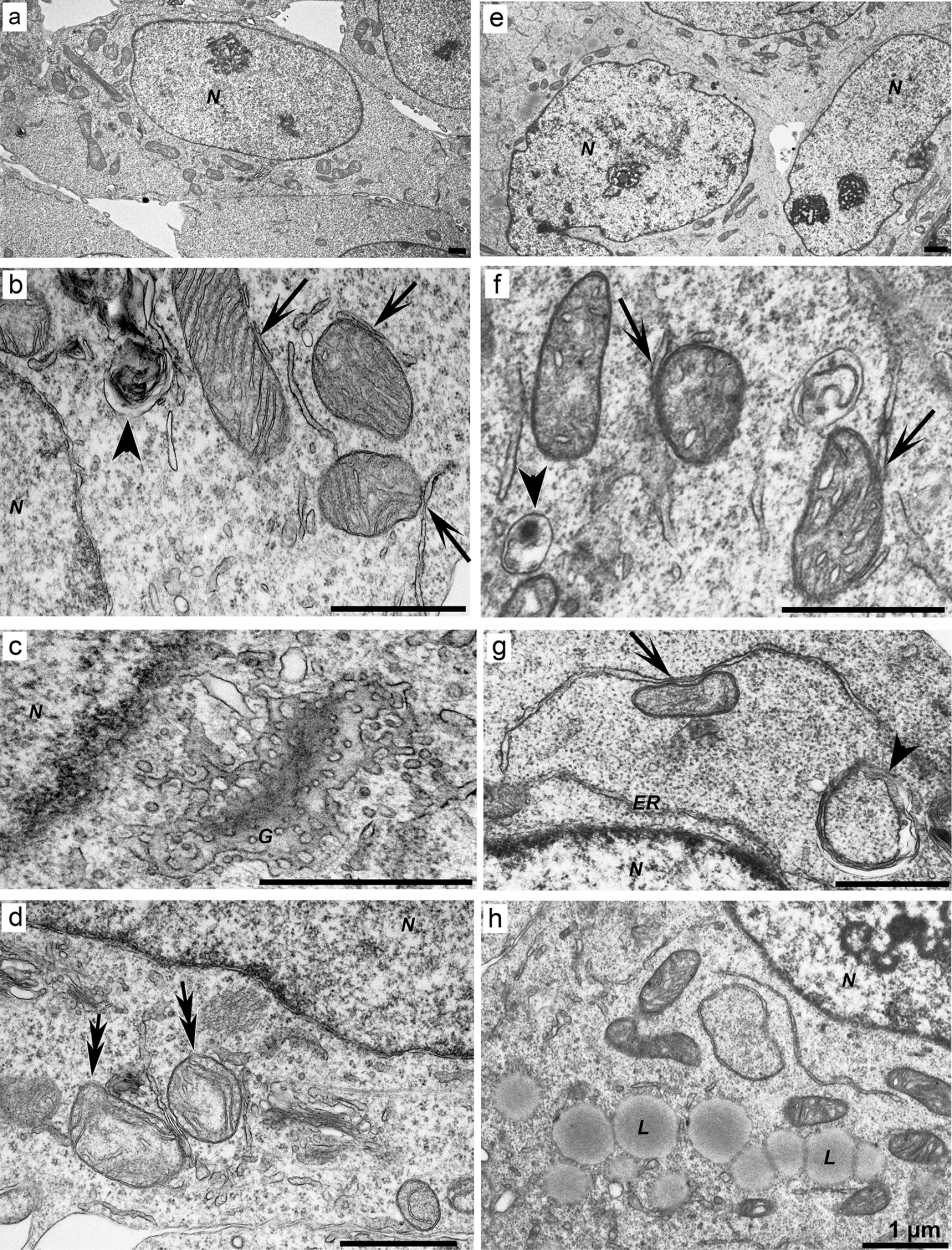
**Ultrastructure of mutant cells with a deletion (clone 8D, panels a–d) or with an ORF shift (clone 8H, panels e–h) in the huntingtin gene.** (a), (e) An overview of the cells. (b) Mitochondria with narrow cristae; the *arrowhead* marks an autolysosome. (c) A well-developed Golgi complex (*G*). (d) Defective mitochondria with a light, transparent matrix (marked with *double arrows*). (f) Mitochondria with a dense matrix and swollen cristae, in contact with ER membranes, and autolysosomes (marked with an *arrowhead*). (g) Long membranes of the ER (*ER*) near the cell nucleus; the *arrowhead* marks a large lysosome. (h) Accumulation of lipid bodies (*L*) in the cytoplasm. Contacts of mitochondria with ER membranes are indicated with *arrows*. *N*: nucleus. *Scale bars*: 1 μm.

The cells of clones 8D and 8Hhad similar shapes and structures of the nuclei as compared with the original control cells (Fig 5A and 5E). Both types of mutant cells are characterized by the presence of mitochondria with a light or dense matrix, respectively (Fig 5B and 5F), autolysosomes or autophagosomes (Fig 5B, 5F and 5G), and a well-developed Golgi apparatus (Fig 5C). Short and elongated rough ER cisternae contacting certain mitochondria in the cells of both clones were also observed. In some cells of both clones, mitochondria with a sparse (rarefied) matrix and dislocated cristae were identified (Fig 5D), and accumulation of lipid droplets was detected in the cells of clone 8H (Fig 5H).

### Ultrastructure of the mutant cells harboring the *HTT* gene with an insertion of 100–150 CAG repeats (clone 6H)

**Fig 6:**
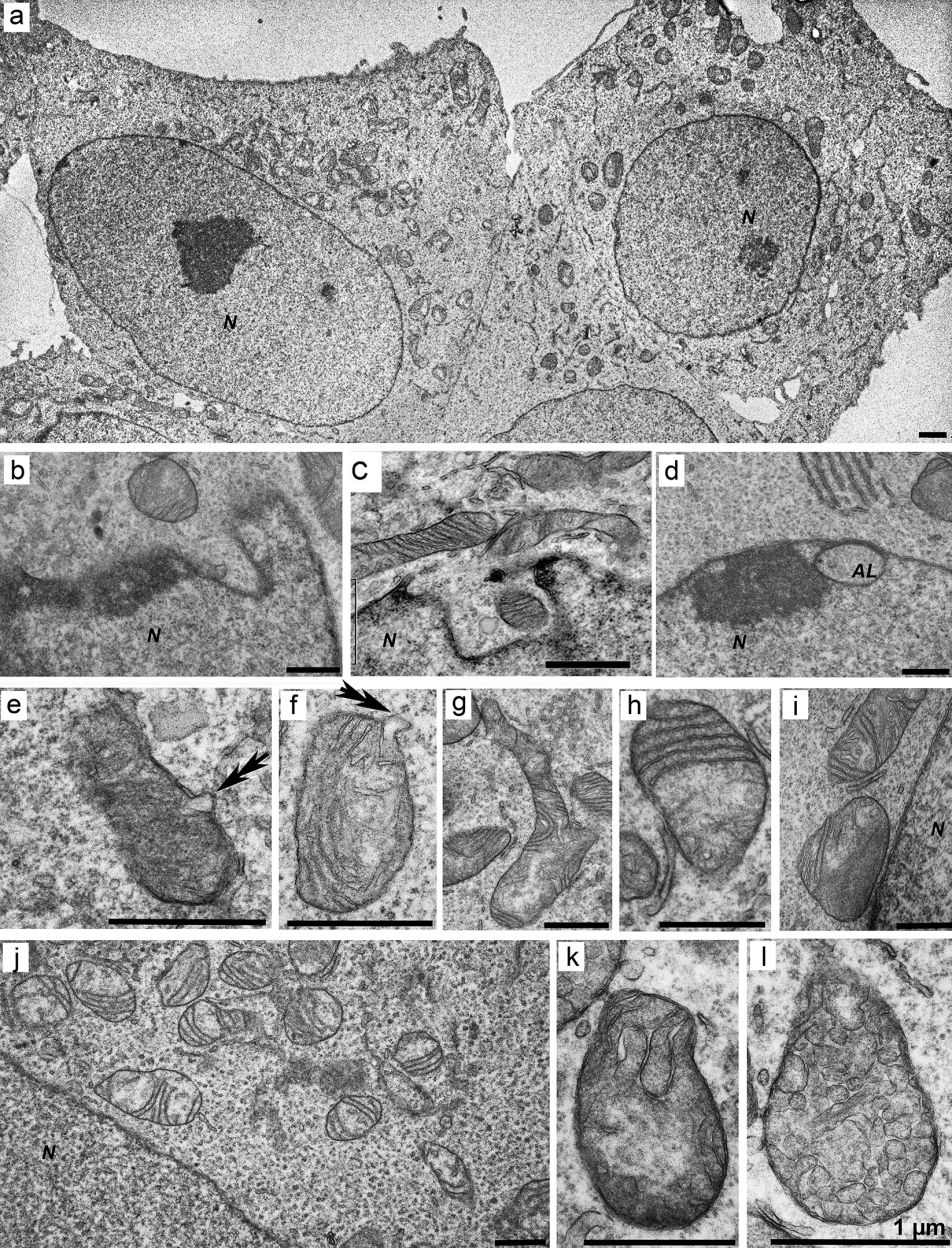
Morphology of cells of mutant clone 6H with a single insertion of 100–150 CAG repeats into the huntingtin gene. (a) An overview of the cells. (b–d) Invagination of the nuclear envelope and annular membrane incorporation (*AL*) into the nucleoplasm. (e and f) A disturbance in the structure of the mitochondrial membrane (*double arrows*). (g) Mitochondria of unusual shape. (h and i) Partial absence of cristae in the mitochondria with low density of the matrix. (j) A group of mitochondria with low density of matrix. (k and l) Mitochondria with deformed cristae. *N*: nucleus. *Scale bars*: 1 μm.

**Fig 7:**
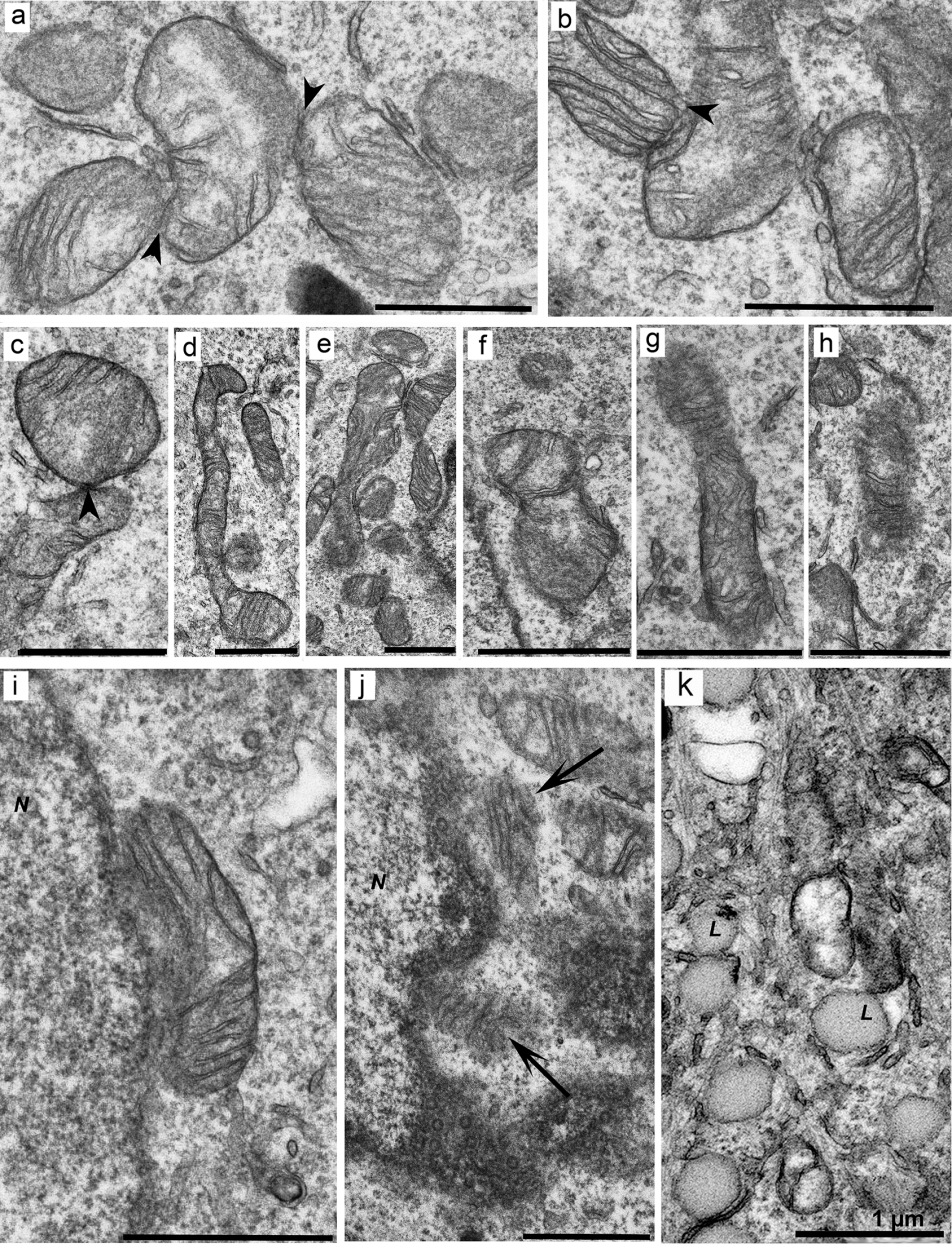
Disturbances of morphology and of contacts between mitochondria in cells of mutant clone 6H. (a–c) Close contacts between mitochondria (labeled with *arrowheads*). (d and e) Lengthening mitochondria. (f) Doubled mitochondria. (g and h) Mitochondria with a partially or completely destroyed envelope. (i and j) Perinuclear location of disintegrating mitochondria (*N–*nucleus). (k) Accumulation of lipid bodies (*L*) near defective mitochondria. *N*: nucleus. *Scale bars*: 1 μm.

The cells often had irregular shapes, contained large round or ellipsoidal nuclei, and were characterized by higher density of organelles in the cytoplasm as compared to the HEK293 cell line (Fig 6A). In some cells, invaginations of the nuclear envelope and annular membrane inclusions in the nucleoplasm were observed (Fig 6B–6D). Many mitochondria had different morphological disturbances: protrusions of the outer membrane (Fig 6E and 6F), changes in the shape of these organelles (Fig 6G), the absence of cristae in large parts of the matrix (Fig 6H and 6I), and the appearance of low density in the matrix (Fig 6J). Significant deformation and degradation of mitochondrial cristae were also observed (Fig 6K and 6L). A distinctive feature of this cell clone was the close contact between defective mitochondria (Fig 7A–7C) and the presence of elongated or “doubled” mitochondria (Fig 7D–7F). Partly fragmented mitochondria or those completely lacking the envelopes (Fig 7G and 7H) were often located in the perinuclear region (Fig 7I and 7J). Furthermore, defective mitochondria were also detected in clusters of lipid droplets associated with the membranes of the smooth ER (Fig 7K).

**Fig 8:**
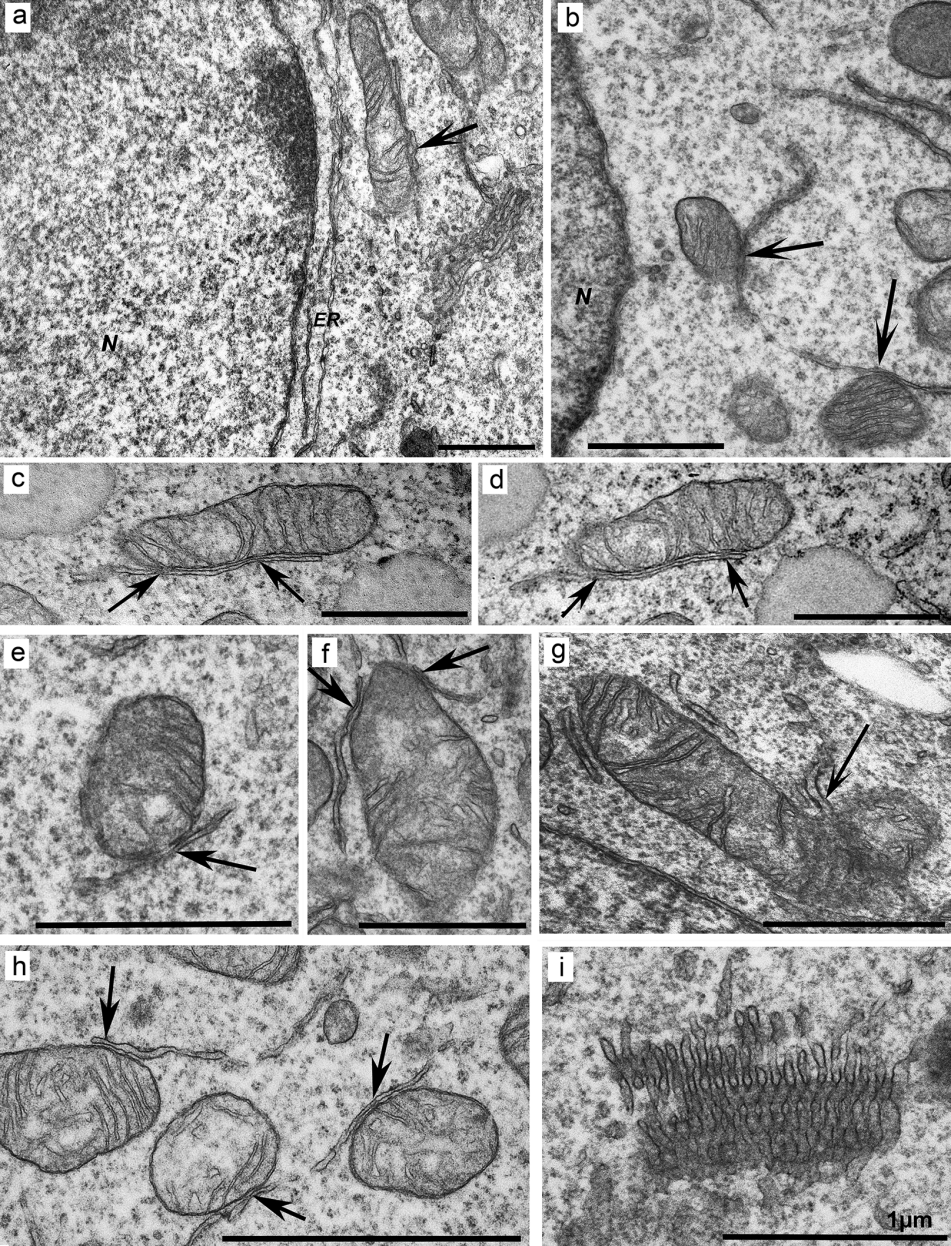
Contacts of ER membranes with mitochondria in mutant cells of the 6H clone. (a) Elongated ER cisternae (*ER*) near the nuclear envelope. (b) Contact of a long ER cisterna with two mitochondria. (c and d) Serial sections of a contact of long ER cisternae with mitochondria (*arrows* mark tight contacts of ER with mitochondria). (e) Contact of two short ER membranes with defective mitochondria (*arrow* marks the contact site). (f) Low density of the mitochondrial matrix near the site of a mitochondrial contact with the ER. (g) A region of the ER near the site of contact with two defective mitochondria (indicated with an *arrow*). (h) Contact of mitochondria showing different degrees of disturbances with ER membranes. (i) An annulate lamellae–like structure with tubular–vesicular organization. *N*: nucleus. *Scale bars*: 1 μm.

**Fig 9:**
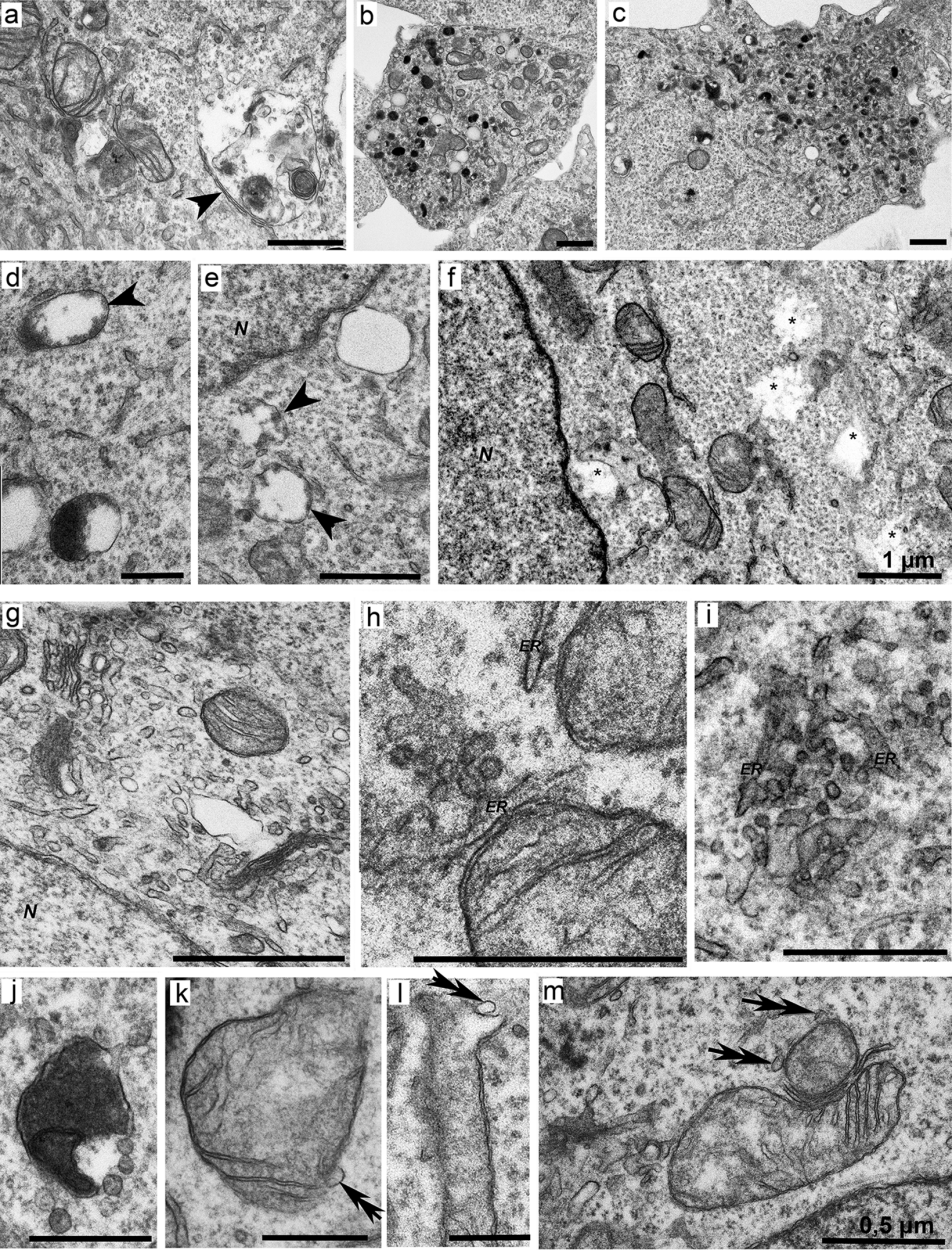
Ultrastructure of lysosomal bodies in cells of mutant clone 6H. (a) A large autolysosome near defective mitochondria (*arrowhead*). (b and c) Clusters of lysosomal components in the cytoplasm of cells. (d and e) Vacuolated lysosomes with light contents and breaches in the membrane (*arrowheads*). (f) Light inclusions not limited to the membrane in the cytoplasm of the cell (*asterisks*). (g) Formation of small vesicles and light vacuoles in the Golgi complex area. (h and i) Clusters of small vesicles near ER membranes. (j) Separation of vesicles from an autolysosome. (k–m) Contact of small vesicles with the envelope of defective mitochondria (indicated by *double arrows*). *N*: nucleus. *Scale bars*: 1 μm (a–f) and 0.5 μm (g–m).

The cells were also characterized by the presence of both long and short cisternae of the rough ER that were closely in contact with normal (Fig 8A and 8B) and defective mitochondria (Fig 8C–8F). The distances between the membranes of the organelles in contact (gap) varied from 6 to 30 nm. Typical and unusual shapes of the annulate lamellae were often observed in the cytoplasm (Fig 8G). A distinctive feature of the 6H cell clone was accumulation of early/small (less than 0.6 μm) and late/large (0.7–2.0 μm) autolysosomes, forming clusters in the cytoplasm of certain cells (Fig 9A–9C). Many large autolysosomes contained dense content adjacent to an empty electron-lucid vacuole (Fig 9D) and often showed disruption of the integrity of the membrane (Fig 9E). Additionally, light inclusions of irregular shapes, without a limiting membrane and similar in size to these autolysosomes, were found in the cells (Fig 9F). Furthermore, the cytoplasm was enriched in vesicles with a diameter of approximately 40–50 nm, observed presumably in the Golgi complex (Fig 9G) as well as near ER membranes (Fig 9H and 9I) and in the vicinity of autolysosomes (Fig 9J). Notably, vesicles of similar size were frequently in contact with the outer membrane of defective mitochondria (Fig 9K–9M). Autophagosomes were randomly present in cells.

### Morphometric analysis of autolysosomes and mitochondrial parameters in HEK293 cells and in cells of different mutant clones

**Fig 10:**
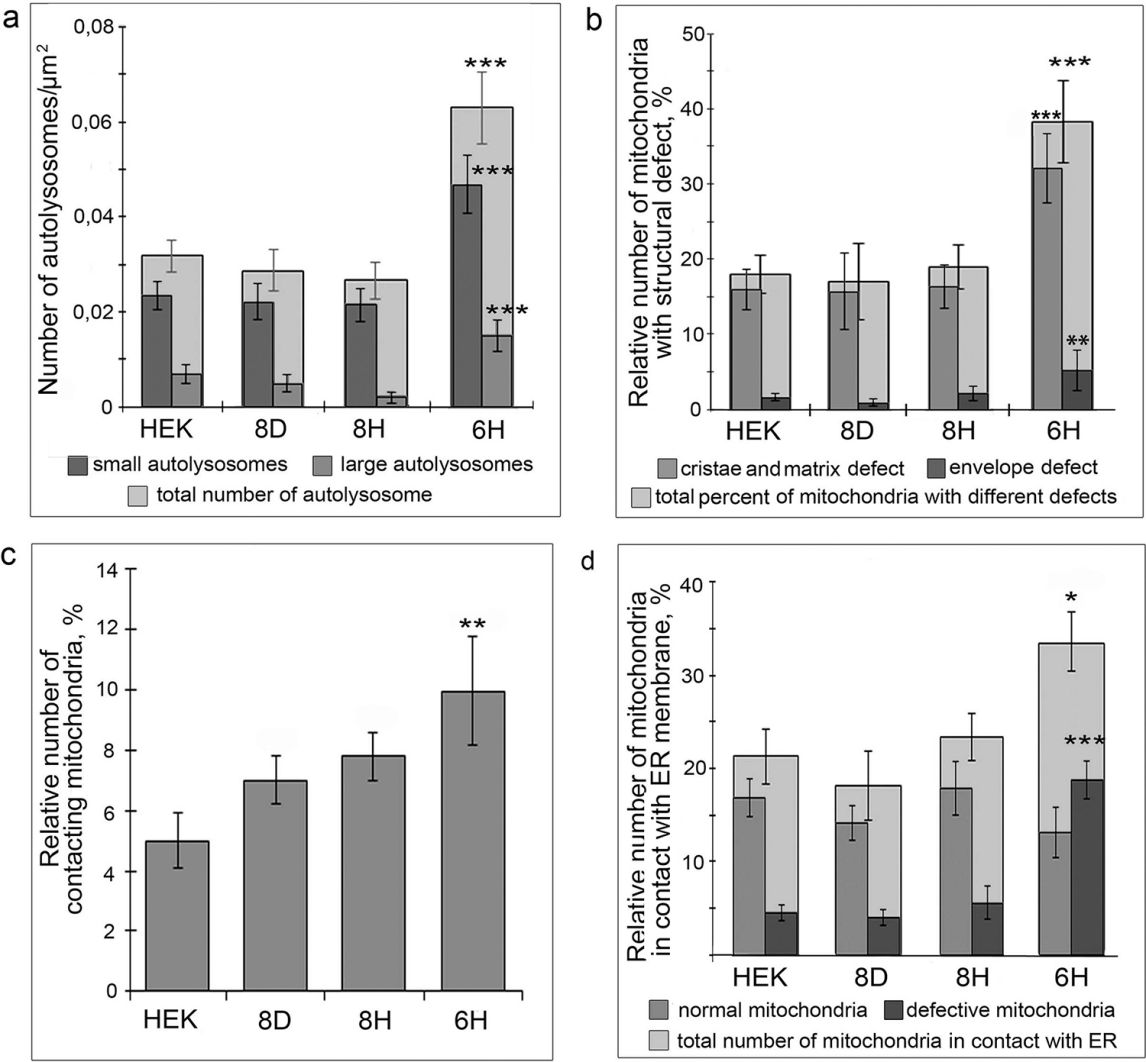
Morphometric analysis of the numerical densities of autolysosomes and mitochondrial parameters in HEK293 cells and in mutant clones with a deletion in the HTT gene, an ORF shift, or addition of CAG repeats. (a) The number of small (diameter up to 0.6 μm) and large (diameter more than 0.7 μm) autolysosomes per μm^2^ of the cytoplasm. (b) The percentage of defective mitochondria with the altered crista shape and orientation in a low-density matrix or envelope defects as a proportion of all mitochondria on a cell section. (c) A relative number of contacting mitochondria among all mitochondria in the cell. (d) The percentage of mitochondria in contact with ER membranes among all mitochondria in the cell. *Error bars* show standard error. *A significant difference in comparison with the control, **p < 0.01; ***p < 0.001.

According to the morphometric analysis, the relative number of small (early) autolysosomes, up to 0.6 μm in diameter, is significantly higher in comparison with that of these organelles having a diameter of 0.7–2.0 μm (late autolysosomes) in all the cell clones studied. Notably, the numbers of small and large autolysosomes, as well as their total number in the cells of the 6H clone doubled as compared to the HEK293 line. The number of small autolysosomes per 1 μm^2^ of the cytoplasm was estimated as 0.023±0.003, 0.022±0.003, 0.021±0.003, and 0.04±0.006 for HEK293 cells and in clones 8D, 8H, and 6H, respectively (Fig 10A). The number of large autolysosomes per 1 μm^2^ of the cytoplasm was estimated at 0.006±0.002, 0.004±0.001, 0.002±0.001, and 0.015±0.003 in HEK293 cells and in clones 8D, 8H, and 6H, respectively. It was found that the proportion of small autolysosomes exceeded the relative number of large organelles 3.2-, 4.4-, 10.7-, and 3.1-fold in the HEK293 line and cell clones 8D, 8H, and 6H, respectively. The most noticeable differences were found in the cells of the 6H clone, where the numbers of small and large autolysosomes as well as their total number significantly increased (doubled) as compared to the HEK293 cell line. At the same time, the proportion of cells containing large autolysosomes increased in clone 6H in comparison with all the others (7% in clone 6H and 3% in clones 8D and 8H and in the control line). Nevertheless, the volume density of large autolysosomes did not significantly differ between cells of clone 6H and cells of the HEK293 line (0.09±0.03 and 0.08±0.03 μm^2^/μm^2^, respectively). This finding suggests that the mean size of large autolysosomes in cells of clone 6H is less than that in cells of the HEK293 line.

Analyses of the mitochondrial fraction with various morphological defects, such as the change in the shape of cristae, low density of the matrix, and disruption of the envelope structure, showed the presence of similar organelles in the cytoplasm of cells in all the clones. Nonetheless, their relative numbers were different. We found that matrix alterations were always accompanied by the cristae disturbances. Therefore, only the numbers of mitochondria with these two types of defects were estimated. The relative numbers of such organelles in the cells of the HEK293 line and in clones 8D, 8H were similar and amounted to 16.1%, 15.9%, and 16.5%, respectively (Fig 10B), whereas the relative numbers of mitochondria with the envelope defects constituted respectively only 1.8%, 1.6%, and 2.31% of all mitochondria on cell sections. Cells of the 6H clone showed 5.5% of mitochondria with envelope defects and 37.8% of defective mitochondria; these mutant cells differed significantly (a 2.1-fold increase) from HEK293 cells in the proportion of mitochondria with all the above defects (Fig 10B).

A comparative analysis of the relative numbers of mitochondria with a contact uncovered a significant increase in the number of such organelles (1.4-, 1.6-, and 2.0-fold) relative to the control in cell clones 8D, 8H, and 6H, respectively (Fig 10C). Contacts of mitochondria with ER membranes could be seen among organelles with normal or defective structure. The proportion of normal mitochondria was higher than that of defective ones 3.7-, 3.5-, and 3.2-fold in cell clones HEK293, 8D, and 8H, respectively (Fig 10D). Whereas a significant increase (3.8-fold) in the proportion of ER-contacting defective mitochondria was recorded in 6H cells, there was a 1.8-fold increase in the total relative number of mitochondria in contact with the ER as compared with the control cells. In addition, the ratios of relative volumes of large autolysosomes in 6H cells and HEK293 line were similar (0.09±0.03 and 0.08±0.04, respectively), whereas the mean diameter of large autolysosomes in this mutant was lower than that in control cells (1.6±0.12 and 2.36±0.39 μm, respectively) (S1 Table).

## Discussion

In this article, we presented a detailed description of the ultrastructural phenotype of three *HTT* mutant cell clones obtained by genome editing: a deletion mutant (clone 8D), an ORF shift mutant (clone 8H), and an insertion mutant with 100 and 150 CAG repeats (6H clone). The isogenic cell model of HD offers an opportunity to study molecular pathogenesis and to test drugs in the presence of an adequate healthy isogenic control that allows us to exclude the influence of genomic variation. In spite of the abnormal karyotype of HEK293 cells, we assumed that mutant-HTT expression should affect the cells and that mutant cell clones should recapitulate some molecular mechanisms of mHTT action because the HD-causing mutation is known to be dominant [1]. Expression of the *HTT* gene was detected in both mutant and original cell lines, even though no mHTT protein aggregates were found in the 6H cell clone harboring mutant *HTT* alleles with expanded CAG repeat tracts (S1 Fig). The evidence of intracellular protein aggregates with the expanded polyglutamine tract has been previously obtained in knock-in PC12 cells with the *HTT*exon1–polyQ transgene that expressed a low-molecular-weight HTT fragment [25]. The absence of protein aggregates is supported by recent findings that formation of misfolded protein plaques is an age-dependent process that occurs only in postmitotic neurons [26]. A similar cellular HTT localization pattern was described in COS cells as a model of HD [27]. Nevertheless, our results suggest that mutant clones manifested enhanced apoptosis and a decreased proliferation rate as compared to an isogenic control (Fig 3).

According to our study, when the number of *HTT* repeats in the cells was expanded, invaginations of the nuclear envelope and disturbances of the mitochondrial structure became more frequent. At the same time, increased contacts of mitochondria with membranes of the rough and smooth ER, fusion of mitochondria, and arise in the number of autolysosomes, vacuolation, and disturbances in the integrity of their membranes, were observed. A knockout or a shift of the reading frame of the *HTT* gene did not cause such changes in the morphology of mutant cells.

### Defects of mitochondria in cells harboring the *HTT* gene with an increased number of CAG repeats

Beyond energy production, mitochondria are involved in the regulation of many processes in the cell: nuclear gene expression, Ca^2+^ homeostasis, cell stress, apoptosis, and necrosis; therefore, their normal structural organization, dynamics, and biogenesis are especially important for the effective functioning of brain nerve cells [12, 28]. According to our morphometric analysis, the total number of mitochondria containing various morphological defects varied from 16.9% to 18.9% of the entire population of such organelles in cells of the original HEK293 line with normal expression of the *HTT* gene and in both mutant clones with the deletion (clone 8D) or an ORF shift (clone 8H). We can speculate that the presence of such mitochondria in cultured cells is related to the process of mitochondria biogenesis, probably owing to their subsequent removal by autophagy [29].The proportion of defective mitochondria grew up to 38% in mutant 6H cells harboring an expanded CAG tract, as compared to the original cell line and the other mutant lines. This alteration apparently causes substantial dysfunction of these organelles. We believe that the accumulation of complex pathological changes in mitochondria included anomalous localization and changes in the shape of cristae as well as their partial absence, low density of the matrix of these organelles, and appearance of deformation in the envelope, probably reflecting the initial stages of their disintegration. Similar mitochondrial defects have been observed previously, in a study on the morphology of spiny neurons derived from iPSCs created from fibroblasts of HD patients with a short (42–47) tract of CAG repeats in the *HTT* gene [13]. Besides, it has been shown that N-terminal mutant HTT fragments that are associated with mitochondria in the brain of Hdh (CAG) 150 knock-in mice affect the organelle trafficking, and this problem progresses with age and disease development [3]. Pathological changes in the structure of mitochondria have also been described in a study on neurons obtained from brain biopsies [30], in patient-specific fibroblasts and myoblasts [31], in postmortem sections of brain cells of HD patients [32], and brain cells of transgenic mice modeling HD (150 CAG repeats in the gene) [33].

We suppose that the emergence of defects in the fine organization of mitochondria correlates with a decline in the glucose metabolism in brain cells [34, 35] and a decrease in respiratory-chain activity in mitochondria isolated from brain cells of patients with HD [36]. Moreover, mutant huntingtin can cause mitochondrial dysfunction, which affects the activity of transcription factors regulating the expression of mitochondrial proteins in the nucleus of the cell [37], whereas aggregates of the mutant huntingtin protein are capable of destroying the nuclear envelope [38]. Our experiments showed that in 6H mutant cells, strongly disrupted mitochondria were often present in close proximity or in contact with the nuclear envelope, and this arrangement probably influences the function of this membrane compartment.

Similarly defective nuclei and swollen mitochondria have been identified in neurons of the striatum of transgenic mice [33]. It has also been found that aggregates of the mutant huntingtin protein destroy the nuclear envelope of the cell [38]. Therefore, it is likely that the observed close association of disintegrated mitochondria and nuclei in the mutant cells with an expanded length of the CAG repeat tract are similar to those found in brain neurons in HD and are probably related to the disruption of the energy metabolism of cells [39]. Besides, our findings are consistent with the results of evaluation of the proliferation rate and the level of apoptosis of the mutant clones. These data suggest that mutations in the first exon of the *HTT* gene in HEK293 cells affect cell viability but do not cause cell death [24]. Overall, our quantitative electron microscopy analysis showed relative contribution of different defects of mitochondrial substructures to the response of HEK293 line cells to expansion of CAG repeats in the *HTT* gene.

### Possible significance of mitochondrial contacts with the ER in mutant cells harboring the *HTT* gene with an increased number of CAG repeats

In recent years, a lot of attention was given to the functional role of mitochondrial contacts with ER membranes called MAMs (mitochondria-associated membranes) related to the transfer of ions, lipids, and Ca^2+^ to mitochondria; these factors may substantially influence organelle properties [40]. It has been demonstrated that dysfunction of such contacts leads to the development of neurodegenerative diseases [11, 41]. We revealed a 3.8-fold increase in the proportion of defective mitochondria that are in contact with the membranes of rough and smooth ER in the cells of clone 6H, which carries the insertion of 100 and 150 CAG repeats into *HTT* in comparison with the control HEK293 cell line. According to literature data [42], we supposed active participation of the ER in the increased proportion of defective mitochondria in the 6H cell clone, possibly owing to dysfunction of MAMs. It has been reported that neuronal death in HD is caused by an increased calcium ion concentration in the cytosol and a disturbance of the calcium concentration in mitochondria [43, 44]. We believe that the increase in the number of mitochondria in contact with ER cisternae in the mutant cells could facilitate the transport of calcium ions to the organelles and disrupt their balance between the organelles and cytosol. This notion is consistent with the data of other investigators who analyzed mitochondrial organization in the cells harboring mutant huntingtin [41, 42].

Besides the important role in the biogenesis of mitochondria, the ER participates in the processes of mitochondrial fission and fusion [45]. Our experiments revealed an increase in the relative number of interacting mitochondria often associated with a low density of matrix in the cells of mutant clones 8D and 8H, and especially in cells of the 6H clone. Given that short ER membranes were observed in the vicinity of a region of contact with mitochondria, we propose that they are in some way involved in this process. We detected the amplification of annulate lamellae as well as their modification called “TUHMAs” (tubulohelical membrane arrays) with an aberrant organization [46]. Therefore, we believe that these alterations may reflect the increase of the lipid synthesis in mutant cells. This finding is consistent with data showing that lipid accumulation is a defining feature of mutant HdhQ111 striatal progenitor cells culture [47]. In our study, we observed contacts of defective mitochondria with 40–50 nm vesicles derived from the ER. We can speculate that this phenomenon may reflect the process of transfer of metabolites to the mitochondria not only via their direct interaction with ER cisternae but also via similar vesicles. Nevertheless, we cannot rule out that these vesicles represent early lysosomes.

### Alterations of the lysosomal system in mutant cells harboring *HTT* with an increased number of CAG repeats

The maintenance of a balance between biogenesis and degradation of mitochondria and other organelles in nerve cells is provided by components of the lytic system including lysosomes, autolysosomes, and autophagosomes and it is one of the important conditions for their functional activity and survival [48]. It has been shown that HTT is involved in the regulation of various autophagic processes such as autophagosome transport, fusion to lysosomes, abnormal protease cleavage, and an altered post-translational modification [49]. The imbalance in the complex interactions of lytic components accompanies the development of HD and other neurodegenerative diseases [50]. According to our data, cells of the mutant 6H clone are characterized by an increase in the total number of autolysosomes [and especially the early (small) type] per μm^2^ of the cytoplasm on a section. This parameter was twice that of the parental HEK293 cell line. In addition, the lytic organelles of different sizes often formed clusters occupying a large part of the cell. Overall, these data allow us to theorize that the increase in the number of CAG repeats in the *HTT* gene activated the process of autolysosome formation. This finding exactly matches the results of other studies suggesting that the activity of early steps of autophagy during the development of HD in cell models [51] and in mutant neurons obtained from iPSCs of HD patients is higher in comparison with normal cells [13, 52]. In addition, an increase in the concentration of autophagosome- and lysosome-like structures in the cytoplasm was demonstrated in the analysis of postmortem samples from patients with HD [53, 54].

According to our morphometric analyses, the ratio of numerical density of early autolysosomes with diameter less than 0.6 μm to the numerical density of larger autolysosomes (0.7–2.0 μm) in the control and all mutant cells was similar; this result could reflect the dynamics of the lytic components at different stages of autophagy and formation and functioning of these organelles. Whereas relative volumes of large autolysosomes in cells of clone 6H and of the HEK293 line were similar (0.09 ± 0.03 and 0.08 ± 0.04 respectively), their mean diameter in mutant cells was significantly (1.48-fold) smaller than that in control cells. One of possible explanations of this phenomenon is that the process of maturation of large autolysosomes in mutant cells was probably disrupted under the influence of the mHTT protein. It has been demonstrated that the mutant HTT protein inhibits autophagosome–lysosome fusion [55]. Our analysis revealed a large number of defectively vacuolized large autolysosomes as well as the autolysosomes with significant disruption of the integrity of their membrane in cells of the 6H clone. Simultaneously, vacuole-like inclusions without membranes were detected in the cytoplasm, allowing us to assume that they can be derivatives of large autolysosomes, judging by their similar sizes. It has been reported that huntingtin-labeled vacuoles manifest the ultrastructural features of early and late autophagosomes (autolysosomes) in clonal striatal cells culture of the mouse brain [56]. Additionally, huntingtin-positive vacuoles were identified in these cells. On the basis of these and our data, we suggest that an increased number of CAG repeats in the *HTT* gene initiates disturbances of the later stages of autophagy in mutant 6H cells.

## Conclusions

In this study, we generated a novel isogenic HD model based on the HEK293 cell line by means of the CRISPR/Cas9 system. The resultant mutant cells with 150 CAG repeats in *HTT* were found to undergo a wide spectrum of pathological changes that were confirmed by morphometric analysis. This analysis revealed structural defects of mitochondria (cristae, matrix, and envelope) together with proliferation of their contacts with the ER. We can hypothesize that these alterations should significantly disturb the energy-dependent metabolism of cells and the balance of Ca^2+^ between mitochondria and the cytoplasm as demonstrated in other studies on HD [28, 57]. The uncovered accumulation of early and late autolysosomes in mutant cells together with artificial vacuolization of large organelles and disruption of integrity of their limiting membrane in 6H mutant cells strongly correlate with the point of involvement of the HTT protein in stimulation of autophagy in the early period and dysregulation of this process at later stages. Our findings are consistent with studies on the morphology of neurons derived from iPSCs of patients with HD. Meanwhile, the use of isogenic cell lines allowed us to exclude the influence of the genetic background and ensured more accurate comparison of mutant cells’ structures. Thus, we for the first time characterized the HEK293 cell culture model with an expanded CAG tract (in *HTT*) obtained via the CRISPR/Cas9 technology and demonstrated that this model can be useful for research into the molecular mechanisms underlying the pathological function of mutant HTT in the development of HD.

## Supporting information

S1 Fig *HTT* allele lengths and an expression assay. (a) PCR analysis of *HTT* allele lengths in mutant cell clones. (b) *HTT* gene expression in mutant clones estimated by RT-PCR. (c) Western blot analysis of HTT expression in mutant cells. (d) Immunofluorescent analysis of cellular localization of the HTT protein in the mutant clone (6H) and isogenic control (Ph).
(TIF)

S2 Fig **FISH analysis of HEK293 Phoenix cells with the painting probe on the human chromosome 4p arm (panels a and b) and chromosome 4 (panels c and d).** FISH revealed that HEK293 Phoenix cells have two full-length copies of chromosome 4 and a translocation of an additional small fragment of chromosome 4 short arm.
(TIF)

S3 Fig Assessment of the small insertions and deletions (indels) in the first exon of the *HTT* gene by a sequence trace decomposition tool. The red columns indicate statistically significant results of deletion/insertion length in the *HTT* alleles of cell clones 8D (a), 8H (b), and 6H (c). The mutation lengths multiple of 3 maintain the ORF (c), whereas all other mutations cause ORF shifts (a, b, and d).
(TIF)

S1 Table Morphometric parameters of large autolysosomes in HEK293 Phoenix and mutant cells. Relative volume densities of large autolysosomes (max. diameter 0.7–2.5 μm) in control and mutant cells were similar, whereas the maximal diameter of autolysosomes was lower in clone 6H than in HEK293. (SD): standard deviation.
(DOCX)
