# Supplementary figures and images for: Introducing an expanded CAG tract into the huntingtin gene causes a wide spectrum of ultrastructural defects in cultured human cells
Source: PLoS One. 2018 Oct 17;13(10):e0204735. doi: 10.1371/journal.pone.0204735 (PMC6192588; doi:10.1371/journal.pone.0204735)

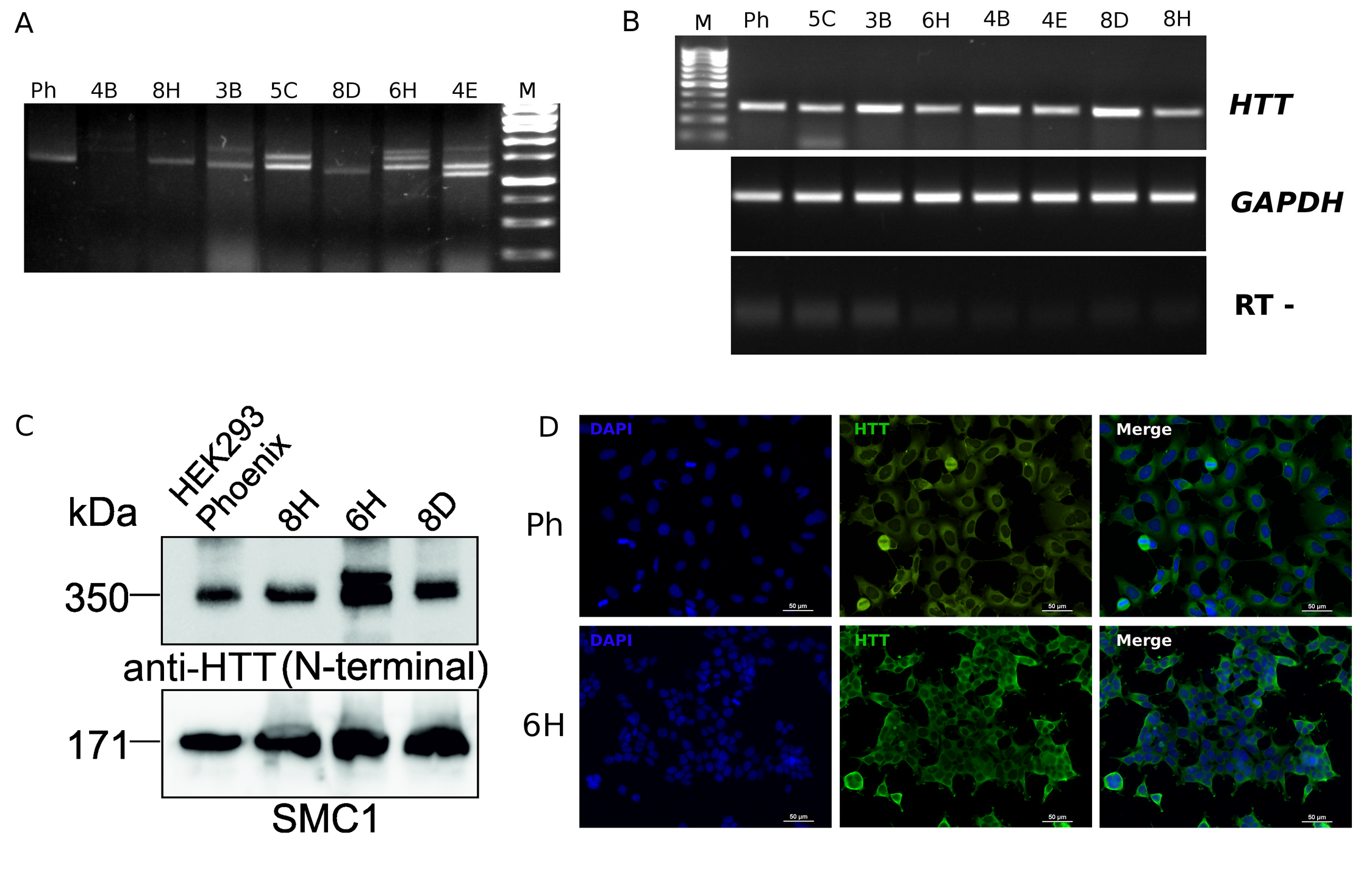

Supplement: S1 Fig — (a) PCR analysis of HTT allele lengths in mutant cell clones. (b) HTT gene expression in mutant clones estimated by RT-PCR. (c) Western blot analysis of HTT expression in mutant cells. (d) Immunofluorescent analysis of cellular localization of the HTT protein in the mutant clone (6H) and isogenic control (Ph). (TIF) [file pone.0204735.s001.tif]

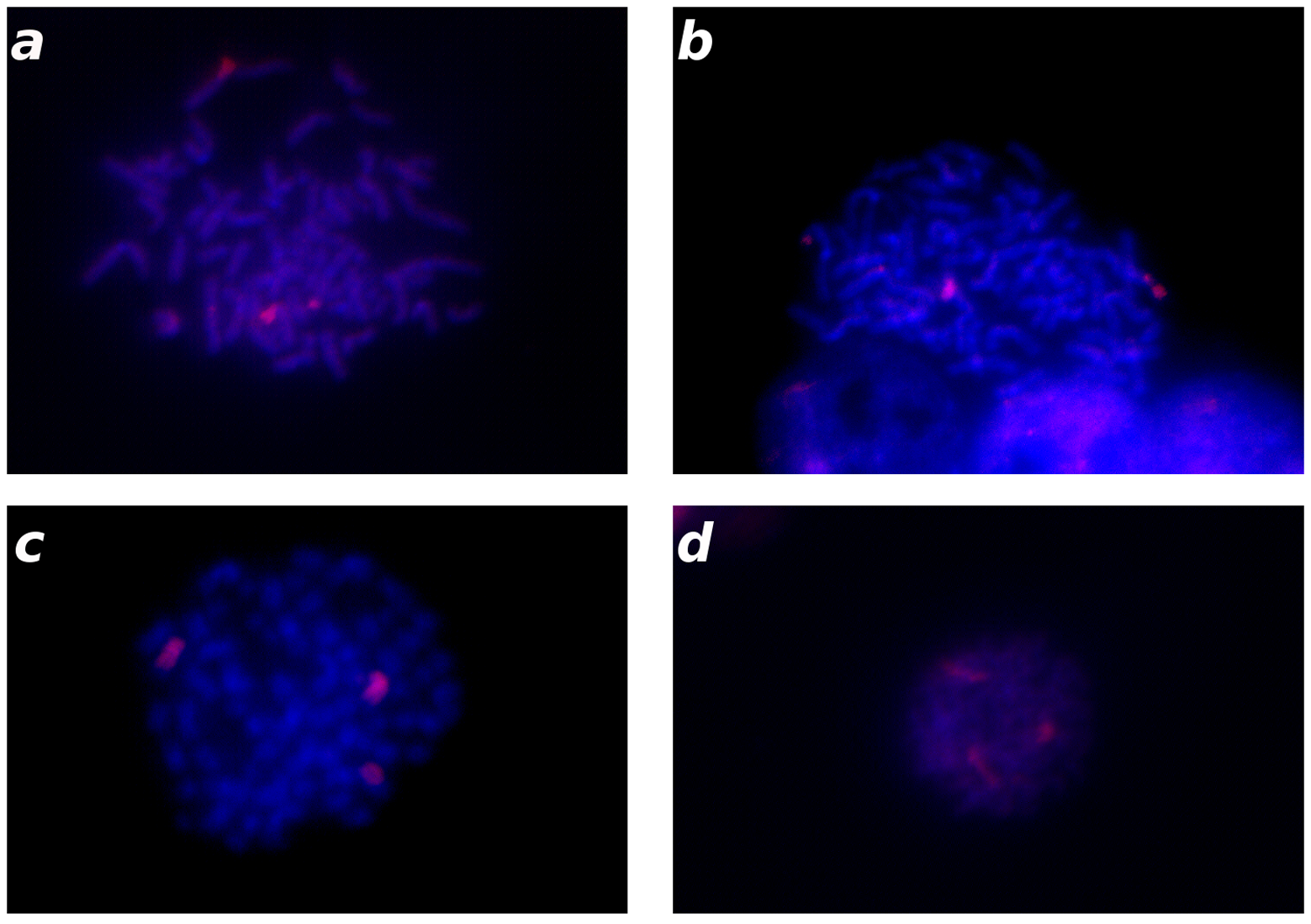

Supplement: S2 Fig — FISH analysis of HEK293 Phoenix cells with the painting probe on the human chromosome 4p arm (panels a and b) and chromosome 4 (panels c and d). FISH revealed that HEK293 Phoenix cells have two full-length copies of chromosome 4 and a translocation of an additional small fragment of chromosome 4 short arm. (TIF) [file pone.0204735.s002.tif]

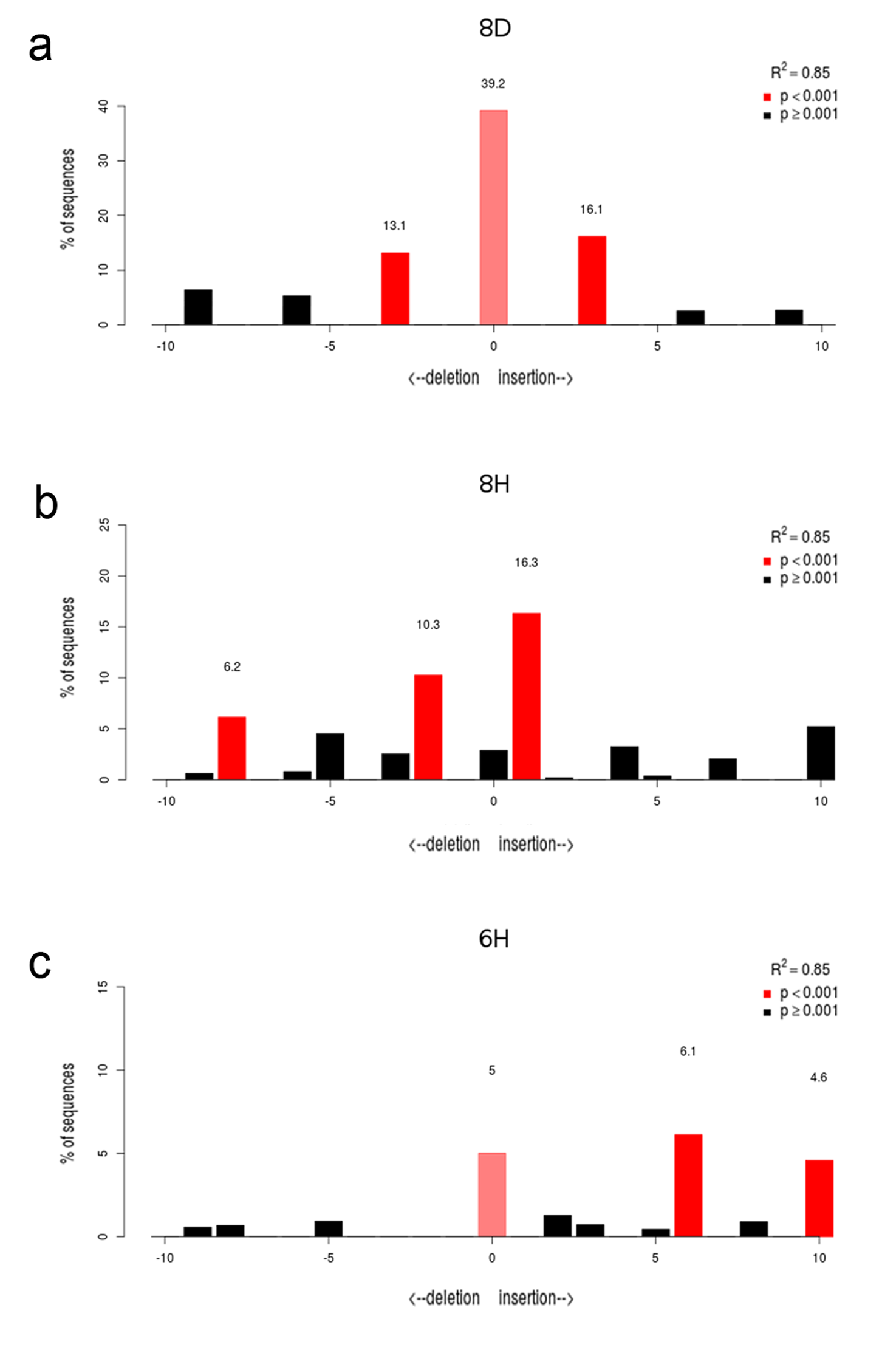

Supplement: S3 Fig — The red columns indicate statistically significant results of deletion/insertion length in the HTT alleles of cell clones 8D (a), 8H (b), and 6H (c). The mutation lengths multiple of 3 maintain the ORF (c), whereas all other mutations cause ORF shifts (a, b, and d). (TIF) [file pone.0204735.s003.tif]
