# Supplementary material for: Introducing an expanded CAG tract into the huntingtin gene causes a wide spectrum of ultrastructural defects in cultured human cells
Source: PLoS One. 2018 Oct 17;13(10):e0204735. doi: 10.1371/journal.pone.0204735 (PMC6192588; doi:10.1371/journal.pone.0204735)
Supplement: S1 Table — Relative volume densities of large autolysosomes (max. diameter 0.7–2.5 μm) in control and mutant cells were similar, whereas the maximal diameter of autolysosomes was lower in clone 6H than in HEK293. (SD): standard deviation. (DOCX) [file pone.0204735.s004.docx]

| Name of normal or mutant cell line | Number of cells analyzed | Relative volume density of autolysosomes, µm^2^/µm^2^ (SD) | Mean maximal diameter of autolysosomes,  µm (SD) |
| --- | --- | --- | --- |
| HEK293Phoenix | 44 | 0.09 (0.03) | 2.36 (0.4) |
| Clone 8D | 35 | 0.08 (0.04) | 1.84 (0.13) |
| Clone 8H | 37 | 0.07 (0.03) | 1.94 (0.14) |
| Clone 6H | 30 | 0.08 (0.03) | 1.60 (0.12) |
